# When two worlds collide: the influence of an obstacle in peripersonal space on multisensory encoding

**DOI:** 10.1007/s00221-021-06072-1

**Published:** 2021-03-29

**Authors:** Rudmer Menger, Alyanne M. De Haan, Stefan Van der Stigchel, H. Chris Dijkerman

**Affiliations:** Experimental Psychology, Helmholtz Institute, Utrecht University, Heidelberglaan 1, 3508 TC Utrecht, The Netherlands

**Keywords:** Visual, Tactile, Multisensory, Reaching, Peripersonal

## Abstract

Multisensory coding of the space surrounding our body, the peripersonal space, is crucial for motor control. Recently, it has been proposed that an important function of multisensory coding is that it allows anticipation of the tactile consequences of contact with a nearby object. Indeed, performing goal-directed actions (i.e. pointing and grasping) induces a continuous visuotactile remapping as a function of on-line sensorimotor requirements. Here, we investigated whether visuotactile remapping can be induced by obstacles, e.g. objects that are not the target of the grasping movement. In the current experiment, we used a cross-modal obstacle avoidance paradigm, in which participants reached past an obstacle to grasp a second object. Participants indicated the location of tactile targets delivered to the hand during the grasping movement, while a visual cue was sometimes presented simultaneously on the to-be-avoided object. The tactile and visual stimulation was triggered when the reaching hand passed a position that was drawn randomly from a continuous set of predetermined locations (between 0 and 200 mm depth at 5 mm intervals). We observed differences in visuotactile interaction during obstacle avoidance dependent on the location of the stimulation trigger: visual interference was enhanced for tactile stimulation that occurred when the hand was near the to-be-avoided object. We show that to-be-avoided obstacles, which are relevant for action but are not to-be-interacted with (as the terminus of an action), automatically evoke the tactile consequences of interaction. This shows that visuotactile remapping extends to obstacle avoidance and that this process is flexible.

## Introduction

In the course of each day, we perform many obstacle avoidance movements. Not knocking into other pedestrians, doorposts and cups of tea are tasks performed with an ease and expertise that betrays the automatic and unconscious nature of what must be a very complex skill. Indeed, to perform avoidance actions nearly all available sources of information—visual, proprioceptive, tactile and audio—must be combined dynamically and in real-time. Therefore, the study of this type of obstacle avoidance behaviour can reveal interesting interactions between these different streams of information and shed light on the information the brain processes in order to program complex movements. That is, the lessons we can learn from obstacle avoidance may allow for a more accurate conceptual understanding of visuomotor control.

It seems logical that your obstacle avoidance behaviour is influenced by what the obstacle is, and where it is. There are indeed a number of studies that show effects of obstacle features on the spatiotemporal characteristics of avoidance movements (Chapman and Goodale 2008, 2010; de Haan et al. 2014; Menger et al. 2012, 2013a, b, 2014; Mon-Williams and McIntosh 2000; Mon-Williams et al. 2001; Rice et al. 2006; Tresilian 1998). The response to obstacle locations has been described as subtle and precise (Mon-Williams et al. 2001), meaning that each individual obstacle location gives rise to a unique avoidance response (see Menger et al. 2014).

While multiple senses are likely to be involved in avoiding collisions with non-target object, most studies have focused on the processing of visual information concerning the obstacle and its incorporation into a motor plan. It is currently unknown how tactile and visual information in an obstacle avoidance situation interact in order to program and control avoidance movements. Moreover, what can these cross-modal interactions tell us about the role of obstacles in the coding of peripersonal space, i.e. the sector of space closely surrounding one’s body? Here, therefore, we are interested in the cross-modal interactions that occur during obstacle avoidance to better understand how peripersonal space facilitates avoidance behaviour.

It is likely that obstacles will activate multisensory networks in the brain because obstacles are—almost by definition—near to the body and, as such, within the peripersonal space of individuals (Rizzolatti et al. 1997). Sensory processing of the peripersonal space is characterised by bimodal neurons in premotor and parietal areas responding both to tactile stimuli on the body as well as to visual stimuli near the body (Duhamel et al. 1998; Graziano et al. 1997; Rizzolatti et al. 1981), which are involved in making visuotactile predictions, i.e. predicting future touch from visual information (Clery et al. 2015; Dijkerman and Medendorp 2021; Kandula et al. 2015; Noel et al. 2018). By using these predictions, motor actions can be prepared according to the desired tactile outcome, for instance catching a ball, or avoiding being hit in the face by it. Thus, peripersonal space processing serves two hypothesized functions (Rizzolatti et al. 1997; Brozzoli et al. 2014; de Vignemont and Iannetti 2015) which are both in line with the function of obstacle avoidance: to successfully guide the hand to a goal object while avoiding non-goal objects, i.e. through efficient voluntary motor control and automatic defensive actions.

To be more specific, the first hypothesized function of peripersonal space is the planning of movements towards items and individuals in the space closely surrounding us and predicting the consequences of these movements. This assumption is based on the research by Brozzoli et al. (2010) who showed that when grasping an object, a flash on one side of a target object improved processing of tactile stimuli on the finger that would touch that part of the object at the end of the grasping movement. This effect was particularly clear during execution of the grasping movement. These findings suggest a dynamic link between visual information on a nearby target object and tactile processing on the approaching hand (Brozzoli et al. 2009,2010). More recently, it has been proposed that expectation effects play a role in peripersonal space coding (Hobeika et al. 2020; Kandula et al. 2017). In particular, peripersonal coding allows anticipation of tactile consequences of contact with a nearby object (Clery et al., 2015,2017; Kandula et al. 2015). Further behavioural evidence comes from demonstrations of visuotactile interactions when objects are near the hand (Spence et al. 2004b; Pavani and Castiello 2004; Brozzoli et al. 2009). These behavioural claims are supported by an fMRI study showing that parietal visuo-tactile cortical areas are activated when objects are near specific parts of a human body (Brozzoli et al. 2011).

Second, objects and people in our close surroundings may also approach us and, as such, may warrant a defensive movement. Facilitation of detection of these objects or people and initiating defensive postures or actions is thought to be another function of multisensory coding in peripersonal space (Rizzolatti et al. 1997; Brozzoli et al. 2014). Evidence for a defensive mechanism comes from monkey studies by Cooke and Graziano (e.g. Cooke and Graziano, 2004; Graziano and Cooke 2006) who found that monkeys executed defensive movements like squinting or blocking when the regions that corresponded with looming or nearby objects were artificially stimulated. Evidence for a similar defensive peripersonal space system in humans comes from the defensive hand-blink reflex, that had been found to be modulated by the proximity of the hand to the face in peripersonal space (Sambo et al. 2012). Furthermore, behavioural studies in humans show that the threatening stimuli extend peripersonal space boundaries (de Haan et al. 2016; Ferri et al. 2015; Kandula et al. 2017; Taffou and Viaud-Delmon 2014).

These studies show spatial visuotactile interactions when some form of contact is expected—either the subject will contact the object or the object will contact the subject. There are, however, situations where objects are relevant for to-be-performed actions but not to-be-contacted, such as in obstacle avoidance. Here we were interested in investigating whether spatial visuotactile interactions could be induced by objects that are not a target of the action, but that are nevertheless relevant when performing the action. This way, we can tell whether predicting the tactile consequences of action includes automatically predicting contact and collisions with all relevant objects even if they are not the terminus of action.

In order to answer this question, we used a cross-modal obstacle avoidance paradigm, in which participants reached and passed an obstacle to grasp a second object. Participants indicated the location of tactile targets delivered to the hand during the movement, while a visual distractor was sometimes presented on the obstacle. Simultaneous tactile and visual stimulation was triggered when the reaching hand passed a position drawn randomly from a set of predetermined locations. Several studies have shown that the cross-modal congruency task can provide an experimental index of common spatial location across different sensory modalities (Spaccasassi and Maravita 2020; Spence et al. 2004b). The cross-modal congruency task can therefore be used to determine the multisensory representation of visuotactile space. As such, this paradigm is suited for investigating the contributions of visual, tactile and proprioceptive inputs to the multisensory representation of peripersonal space.

## Methods

### Participants

Ten participants (5 men and 5 women) volunteered for this study in exchange for curricular credit and gave their informed consent. All participants were right-handed and had normal or corrected-to-normal visual acuity and were naïve as to the purpose of the experiment. Our faculty’s institutional review board under the Medical Research Act ruled that this experiment did not need approval from a Medical Ethics Review Committee. The experiment was conducted according to the guidelines provided by the Helsinki Declaration (WMA 2013).

Sample size was determined using power analysis software, viz. G*Power (Franz Paul, Universität Kiel, Germany). We obtained a partial *η*^2^ from an earlier study (Menger et al. 2012). The effect size, *f*, was determined to be 0.57. This effect size related to the difference in deviation of the hand movement between the target with similar obstacle condition and the target with dissimilar obstacle condition (i.e. a main effect of target-distractor congruency on the deviation of the hand from the obstacle). The effect size of 0.57 will be detected with a precision = *α *0.05 (two-sided) and with = *β* 0.05 (power = 95%). We should, therefore, be able to detect any effect on trajectory with this sample size.

### Materials and apparatus

**Fig. 1:**
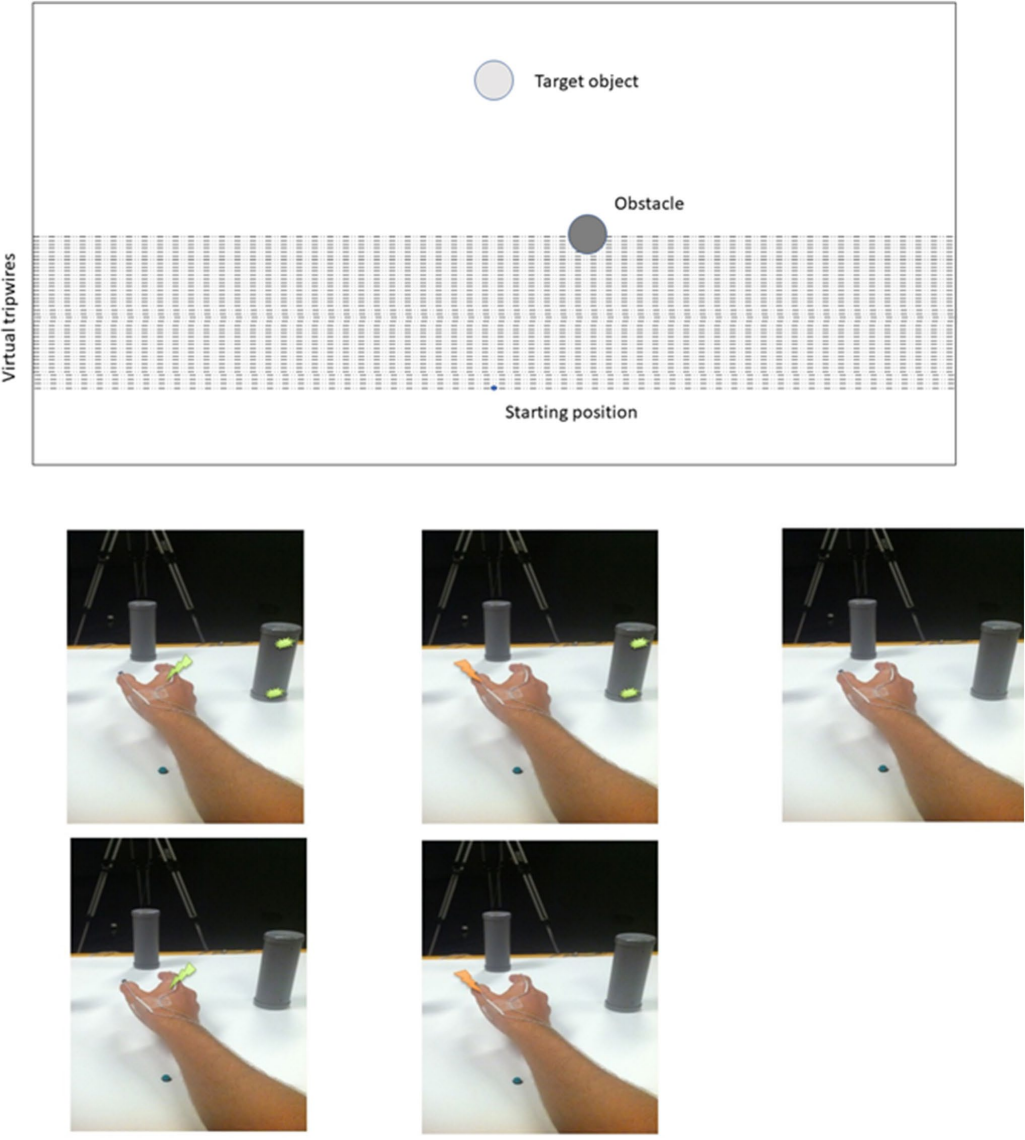
Top: Schematic representation of the experimental setup, showing starting position (small blue circle), the target object (light grey circle) and the non-target object (dark circle). The lines show the locations of the forty virtual tripwires. Bottom: The five different conditions: From left to right: Visuotactile congruent, visuotactile incongruent, control, tactile congruent and tactile incongruent

Participants were tested individually in a quiet dimly illuminated room. They were seated at a white table with two embedded buttons: one start-button, located near the participant, and one target-button located at a reachable depth of 400 mm at 0 mm width, respective to the midline (see Fig. 1). Participants were midsaggitally aligned with the midline of the workspace. Participants had to reach-to-grasp objects while avoiding an obstacle (obstacle avoidance task). During this action unimodal or multimodal stimuli could be delivered, tactile or visuotactile stimuli respectively and participants had to perform a tactile discrimination task.

Hollow plastic cylinders (50 mm and 150 mm height × 50 mm diameter) served as target object and as non-target object. The tall object was the non-target, while the short object functioned as target. The target object was made shorter to prevent problems with occlusion of the infrared motion system markers placed on the hand. The target object was weighted down with sand to ensure participant’s responses were analogue to our earlier experiments with wooden objects. The target button would respond to the object being lifted from it and triggering it would signify the end of a trial. Two red-coloured LEDs were embedded (near the top and the bottom) within the non-target which were facing toward the participant. The LEDs were programmed to emit bursts of light for 30 ms simultaneously. The target object was placed on the target button, while the non-target was always placed at 200 mm depth and 100 mm width to the right of the midline (from the participant’s perspective), see Fig. 1 for a representation of the set up. For right hand reaches, this location ensures that the non-target objects actually act as obstacles to the hand movements. In a control experiment, we placed non-targets at the left of the midline, where no influence of visuotactile interactions is expected, as there is a very low likelihood of touching the non-targets (i.e. they are not obstacles) (Menger et al. 2013a, b).

The tactile stimulation was achieved by use of vibro-tactile motors that presented 180 Hz vibrations for a period of 100 ms to either the index finger or the thumb of the ‘acting’ hand, and stimulation took place during task execution (explained in more detail in the next paragraph). The motors were attached to the middle of the most proximal phalanx of both fingers. Participants were asked to report the location they felt the tactile stimulus by pressing one of two buttons on the response box with their left hand (tactile discrimination task): No explicit instructions were given regarding which fingers of the left hand to use for responding to the tactile stimulus on the right hand. Based on the configuration of the button box and our observations, most participants probably used the index finger and middle finger to respond to the tactile stimuli on index finger and thumb of the right hand respectively.

Movement kinematics of the right hand were recorded using 3D Investigator™ Motion Capture System (Northern Digital, Waterloo, Ontario, Canada) at a sampling rate of 200 Hz. The markers were attached to the tips of the participants’ index fingers and thumbs (see also Mon-Williams and McIntosh 2000) in such a way as to minimize occlusion by objects. Furthermore, by securing the cables to the participants’ arms and hands, great care was taken to avoid interference by the markers itself on the movements. A custom built microcontroller interfaced with the motion capture system and a stimulus computer to allow for real-time integration of kinematic data into stimulus presentation. This setup was made so that we could present LED and tactile stimulation during movement when a certain location was reached. In short, we configured a set of virtual tripwires that automatically and quickly produced stimulation based on marker location. If a tripwire (defined in space as a 2D plane) was triggered then stimulation would occur within 5 ms.

### Design

We used a continuous design, where 40 different locations between the starting location and the non-target object’s midpoint acted as tripwires for stimulation. Tactile stimuli could be presented alone or together with a simultaneous visual stimulus (both LED’s simultaneously for 30 ms) on the non-target object. As the obstacles (and thus the visual stimuli) were always to the right of the reaching arm, and a typical right handed grasp posture will place the index finger on the right as well, the visual and tactile stimuli and therefore the visuotactile trials were considered congruent when the tactile stimulus was on the index finger and incongruent if the tactile stimulus was presented on the thumb. Similarly, for tactile-only trials, tactile stimuli were considered congruent when presented to the index finger and incongruent when presented to the thumb, because the non-target (although not flashing) was always located to the right of the reaching hand. Furthermore, a catch-trial condition was included, where participants never received tactile stimulation during task execution. This was to ensure that participants did not simply wait for the stimulation to occur in order to perform better on the tactile discrimination task. The catch-trials were presented 16 times (10%). The conditions in our design are therefore: *tactile congruent*, *tactile incongruent*, *visuotactile congruent*, *visuotactile incongruent* and *catch*. The total number of trials therefore numbered 40 × 4 + 16 = 176. Five repetitions of each condition were presented as practice trials prior to the experiment.

### Procedure

Participants had to reach-to-grasp objects while avoiding an obstacle. During this action, unimodal (tactile) or multimodal (visuotactile) stimuli could be delivered. They were instructed to have their right thumb and index finger on the starting button in a closed pincer posture until task execution was required. The experiment was self-paced: once participants pushed the starting button, a warning signal would alert them to the start of the trial and after a random interval between 800 and 1200 ms a different auditory signal would prompt them to execute their task. The two tasks were to be performed at the same time: perform a reach to grasp the target object in order to lift it, while also reporting the location of a tactile stimulus on their acting hand. We further instructed the participants to grasp the middle of the target object with their thumb and index finger and react as quickly as possible to the tactile stimulation while also smoothly and quickly reaching for the target object.

### Analysis

We gathered data on measures of response interference in the tactile discrimination task and in the obstacle avoidance task.

One participant had to be excluded, as he did not carry out the obstacle avoidance task as instructed in 41% of trials (in particular: waiting for the auditory signal and making a smooth, quick movement).

We included 9 participants, who each did 160 trials (and 16 catch trials) for a total of 1440. We had to exclude the first two tripwire locations as described (72 trials in total), as well as 54 trials with incorrect responses, 30 missed trials, 28 trials with reaction times > 3 MAD from the median, 7 non-ballistic hand movements, 18 hand movements that started from somewhere else than near the start position, 7 hand movements that were performed before the trial (and thus the movement recording) had started and 6 trials because the marker was lost from view. Therefore, we analysed a total of 1440–366 = 1074 trials.

For the non-catch trials, we analysed %error of responses (number of incorrect responses), absolute reaction time (time between stimulus presentation and response box button press) and the Cross-modal Congruency Effect (CCE; the difference scores between reaction times in congruent and incongruent conditions). Reaction times (RTs) that were more than 3 MAD from the median for a certain participant were excluded (2.1%).

Because of the continuous design we were able to calculate the slope between space (the locations of the tripwires) and RTs as well as CCEs. We performed Repeated Measures Analyses of Variance (RM ANOVA) on these slopes and also on %Error, with within-subjects factors *Modality* (2 levels: tactile, visuotactile) and *congruency* (2 levels: congruent, incongruent). CCE slopes were analysed with a Paired t-test on the effect of *Modality* (tactile, visuotactile).

For kinematic data from obstacle avoidance movement, we looked at the same trials as for the tactile discrimination task data and analysed Peak velocity (the maximum velocity attained during movement), Movement time (the time from movement onset until the end of the reach-to-grasp movement), Deviation at passing (distance between the location of the index finger and the edge of the non-target at the moment the hand passed the vertical position of the middle of the non-target) and Error at passing (the standard error of the mean calculated for the deviation at passing measure across all the repetitions of a condition by a particular participant, i.e. the less error at passing, the more stereotypical a movement). We performed Repeated Measures Analyses of Variance (RM ANOVA) on these measures, with within-subjects factors *Modality* (2 levels: tactile, visuotactile) and *Congruency* (2 levels: congruent, incongruent).

All kinematic analyses were performed on the *x*, *y* and *z* data from the index finger marker. The raw trajectory data of each trial was filtered by using a dual low-pass second-order Butterworth filter with a cut-off frequency of 20 Hz (see also: Mon-Williams et al. 2001; Tresilian et al. 2005). The filtered trajectory data was then normalized using a cubic spline interpolation into 100 samples (see also: Smeets and Brenner 1995; Tresilian et al. 2005).

## Results

### %Error

**Fig. 2:**
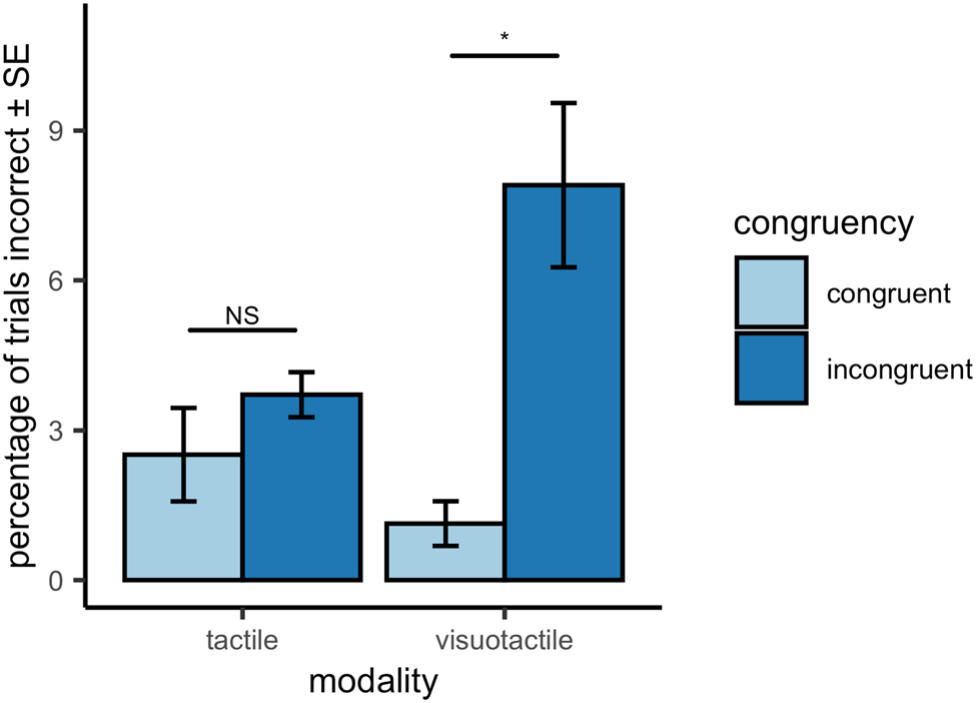
Mean performance scores in %Error across participants for the tactile (left) and visuotactile (right) trials in congruent (light) and incongruent (dark) conditions. Error bars are standard error of the mean

Figure 2 shows the percentage of errors made in tactile discrimination judgments. There was a main effect of congruency on the amount of errors participants made in the tactile discrimination task [*F*(1, 8) = 20.92, *p* = 0.002, *ηp*^2^ = 0.72] and no main effect of modality [*F*(1, 8) = 2.08, *p* = 0.187, *ηp*^2^ = 0.21]. The fewest errors were made in the Visuotactile Congruent condition (1.13 ± 1.34%), then the Tactile Congruent condition (2.51 ± 2.81%) and Tactile Incongruent condition (3.71 ± 1.35%). The most errors were made in the Visuotactile Incongruent condition (7.91 ± 4.94%). We further found an interaction effect between congruency and modality [*F*(1, 8) = *5.63, p* = *0.045, ηp*^2^ = *0.41*] on the percentage of errors made in the tactile discrimination task. Further Bonferroni-corrected (*α* = 0.025) paired t-testing revealed that the amount of errors was higher for visuotactile incongruent condition than for visuotactile congruent condition [*t*(8) = − 3.62, *p* = 0.007, *d* = − 1.71] but not for the tactile only conditions [*t*(8) =  − 1.37, *p* = 0.209, *d* = − 0.64]. This implies that participants made the most mistakes when the non-target object was flashed while the site of tactile stimulation was on the opposite side of the hand. So to summarise, as expected, the amount of errors was larger in the incongruent conditions (tactile and visuotactile) than in the congruent (tactile and visuotactile) conditions, indicating it was harder for participants to judge the site of tactile stimulation when it did not correspond with the side of the hand the non-target object was on. The effect appears to be mostly driven by the visuotactile condition.

### Absolute reaction time

**Fig. 3:**
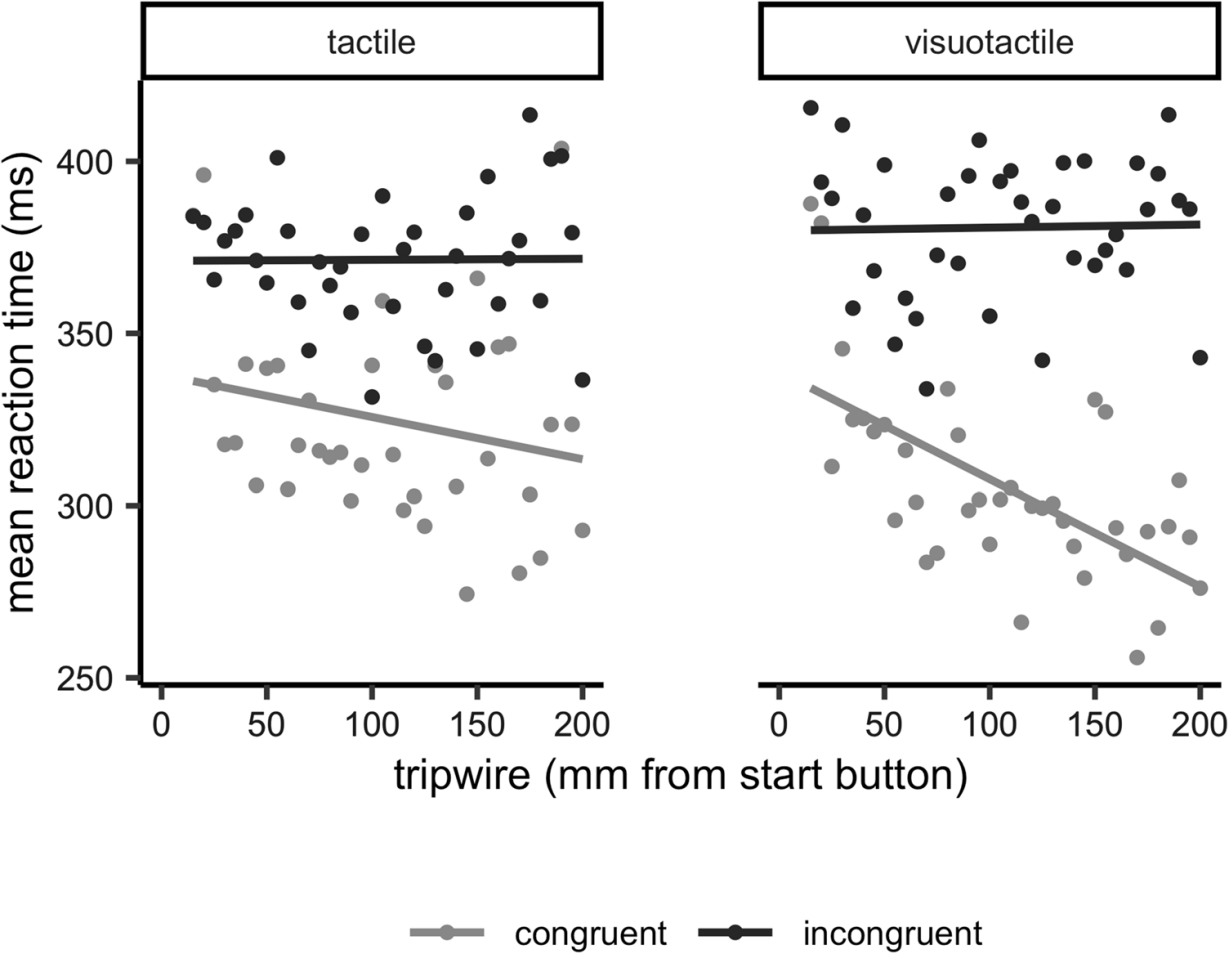
Overview of absolute reaction times in the tactile (left) and visuotactile (right) trials for congruent (light) and incongruent (dark) conditions. The scatter plot represents mean scores for that tripwire location across participants. The lines represent the least mean squares regression lines to show the mean relation between space and absolute reaction time. Please note that the analysis reported in the results section was based on individual slopes

We contrasted space, defined as distance from the start button at which the tactile stimulus was given, with mean reaction times across participants (RTs) in Fig. 3 for all conditions. Over all tripwire locations, average reaction times were lower in congruent (312 ± 110 ms) than in incongruent (375 ± 98 ms) conditions [main effect congruency, *F*(1, 8) = 100.70, *p* < 0.001, *ηp*^2^ = 0.93] but there was no effect of modality and no interaction. As stated in the analysis subsection above, we calculated the slopes of between space and RTs for all participants for each condition. The average of these slopes can be seen in Fig. 3. From this graph, several preliminary conclusions can be made. Most importantly, there seems to be an effect of tripwire location on RTs in the visuotactile congruent condition, but less or none in the other conditions.

Slopes of the fitted linear functions to the data for each condition of each participant showed a main effect of Congruency [*F*(1, 8) = 13.94, *p* = 0.006, *ηp*^2^ = 0.64]. Slopes were more pronounced in congruent conditions than in incongruent conditions. Furthermore, we found an interaction effect between modality and congruency for the individual slopes for space versus reaction times [*F*(1, 8) = 6.52, *p* = 0.034, *ηp*^2^ = 0.45]. Further investigation with Bonferroni-corrected paired *t*-testing (*α* = 0.025) showed that the individual slopes for visuotactile trials differed between congruent and incongruent conditions [*t*(8) = − 5.37, *p* = 0.001, *d* = -2.53, with steeper slopes in the visuotactile congruent than in visuotactile incongruent condition), which was not the case in the tactile only trials [*t*(8) = 0.59, *p* = 0.568, *d* = 0.28]. Individual one sample *t *tests (*α* = 0.0125) showed that the slopes were different from zero only in the visuotactile congruent condition [*t*(8) = − 5.16, *p* = 0.001, *d* = − 1.72] and not in the other conditions [tactile congruent: *t*(8) = − 1.01, *p* = 0.341, *d* = − 0.34, tactile incongruent: *t*(8) = − 1.58, *p* = 0.152, *d* = − 0.53, visuotactile incongruent: *t*(8) = − 0.08, *p* = 0.938, *d* = − 0.03]. Simply put, RTs go down when the hand nears the obstacle, but only when the obstacle is flashed and the tactile stimulation is on the side of the flash. If the tactile stimulation is on the other side or when there is no visual stimulation accompanying the tactile stimulus, the distance between the hand and the obstacle does not modulate RTs.

### Cross-modal congruency effect

**Fig. 4:**
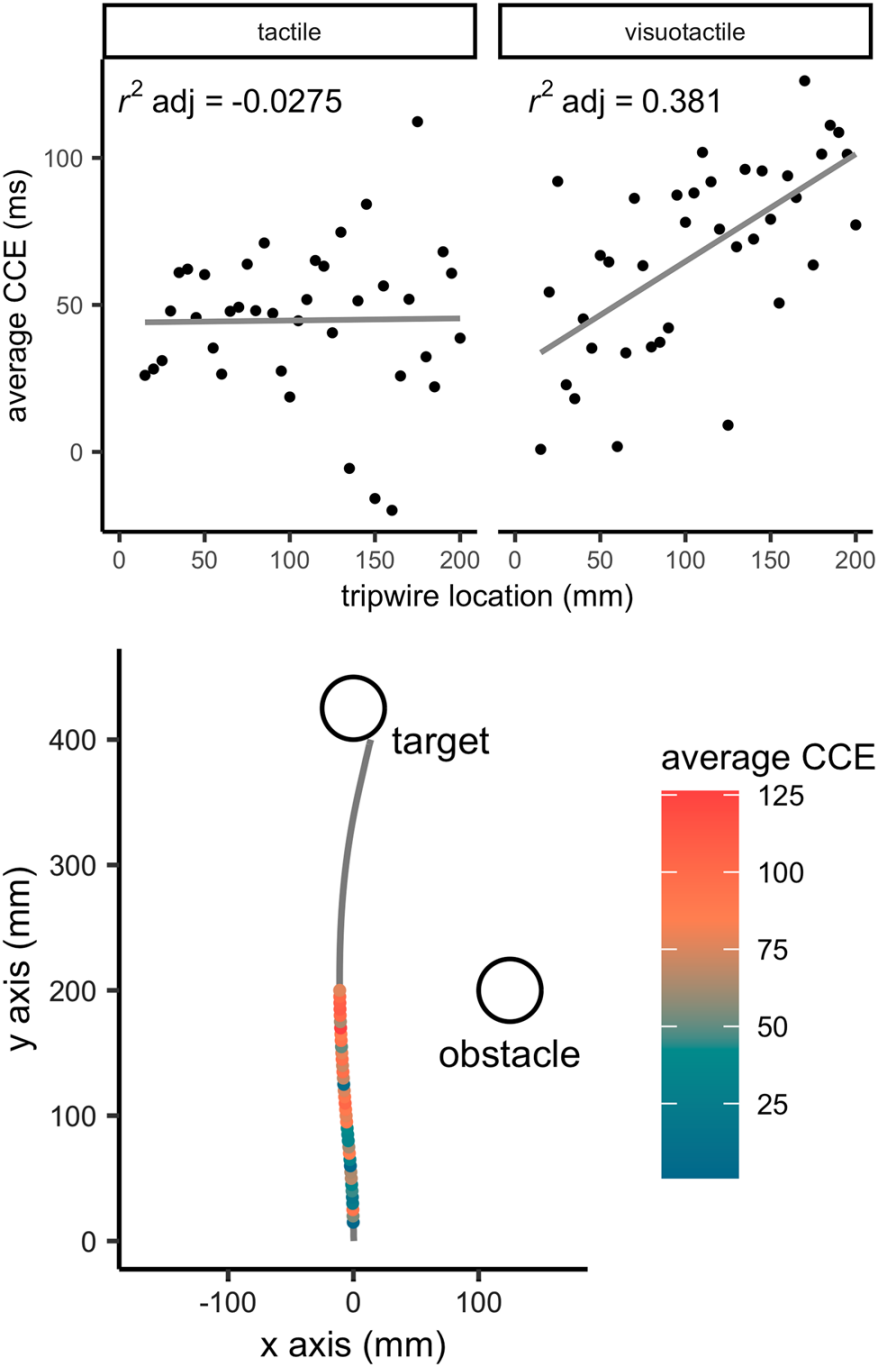
Top Difference scores between participants’ reaction times to valid and invalid stimuli under visuotactile (left Panel) and tactile stimulation (right Panel) across space (cross-modal congruency effect, CCE). The solid lines indicate least mean squares regression lines which shows that space accounts for approximately 38% of variation in the visuotactile conditions, while only for approximately 3% in tactile conditions. Please note that these figures show the group fit for clarity, but analysis was based on individual slopes. Bottom: Top view of a typical hand movement (the average hand movement of all participants in the visuotactile congruent condition) with coloured dots indicating the average visuotactile CCE at each tripwire.

The cross-modal congruency effect (CCE) measure is shown in Fig. 4. This difference score, which was computed between the congruent and incongruent iterations of tripwire stimulations at the same depth, has been known to show the amount of interference offered by multisensory interactions. That is, the valid stimulation conditions exhibit a facilitatory effect on response times, while the invalid stimulation conditions exhibit an inhibitory effect on response times (see also Spence et al. 2004a, b). Although the exact contribution of each factor (valid and invalid stimulation) remains unclear in this metric, the CCE measure does allow for easy and quantified cross-modal comparisons. We have plotted the CCE against space (depth of stimulation or tripwire location from starting position in mm) for both visuotactile and tactile stimulation conditions. The graphs show an apparent effect of space on the visuotactile CCE, while an effect of space on the tactile CCE appears absent. We ran a least mean squares regression on the average CCE’s across participants per stimulation modality and the results showed that our model had an adjusted *r*^2^ of 0.381 in the visuotactile modality condition and an adjusted *r*^2^ of 0.0275 in the tactile condition. We analysed the individual slopes of the relationship between space and the CCE for the visuotactile condition (mean ± SD: 0.39 ± 0.21) and the slopes for the relationship between space and the CCE for the tactile condition (mean ± SD: − 0.09 ± 0.49) with a paired *t *test, which showed a difference between visuotactile and tactile CCE’s [*t*(8) = − 2.23, *p* = 0.029 one-sided, *d* = − 1.05]. Given these data, we can conclude that in the visuotactile condition crossmodal interaction seems to increase when participants’ hands neared the obstacle.

### Kinematic data analysis

For the kinematic data analysis we found no main effects for any measure (Peak velocity, Deviation at passing and Error at passing), except for the movement time measure. For movement time we found a main effect of congruency, *F*(1, 8) = 12.14, *p* = *0.008, ηp*^2^ = *0.60*. This indicates that incongruent trials were performed slower than congruent trials. We found no main effect of modality, but there was a trend for an interaction between modality and congruency [*F*(1, 8) = 4.66, *p* = 0.063, *ηp*^2^ = 0.37]. There was a trend for the difference in movement time between congruent and incongruent conditions to be larger in visuotactile trials than in tactile only trials [Paired *t *test: *t*(8) = − 2.16, *p* = 0.063, *d* = − 1.02].

So, the tactile discrimination task seems to influence total movement time on the hand movements, but not other kinematic aspects. However, this change in movement times does not reflect a trade-off with the tactile discrimination task, as participants were slower in incongruent conditions (similar to the tactile discrimination task), especially in the visuotactile condition.

### Control experiment

In a control experiment, we placed non-targets at the same distance but to the left of the midline, where no influence of visuotactile interactions is expected, as there is a very low likelihood of touching the non-targets (i.e. they are not obstacles, see Menger et al. 2013a, b). Ten right-handed participants volunteered, but the data of three participants had to be excluded: one participant did not carry out the tactile discrimination task at all, one participant started all hand movements before the start of the trial (and thus before kinematics was measured), and one participant had to be excluded as the marker disconnected during the experiment. The procedure was identical to that of the first experiment, except that as the non-target was placed to the left of the midline, tactile stimuli on the thumb were now considered to be congruent and those of the index finger incongruent.

We performed the same repeated measures ANOVAs as in the main experiment and found no significant effects of congruency or modality in the tactile discrimination task on percentage of errors [modality: *F*(1, 6) = 1.93, *p* = 0.214, np2 = 0.24, congruency: *F*(1, 6) = 1.34, *p* = 0.292, *ηp*^2^ = 0.18, interaction: *F*(1, 6) = 0.39, *p* = 0.556, np2 = 0.06) or slopes (reaction times ~ tripwire location) (modality: *F*(1, 6) = 1.53, *p* = 0.263, *ηp*^2^ = 0.20, *F*(1, 6) = 2.05, *p* = 0.202, *ηp*^2^ = 0.26, interaction: *F*(1, 6) = 0.68, *p* = 0.440, *ηp*^2^ = 0.10] and no difference between difference between visuotactile and tactile CCE’s [Paired *t* test: *t*(6) = − 0.28, *p* = 0.787, *d* = − 0.15]. Please note that while a power analysis (G*power) suggests that a minimum of eight participants is required for detecting a difference, a sequential analysis of the Bayes factor in a Bayesian paired *t *test (JASP Team 2020) suggested that increasing the sample size would actually result in increasing evidence that the visuotactile and tactile CCE’s do not differ.

Also, there were no effects on average reaction time over all tripwire locations, nor on the kinematic measures Movement time, Deviation at passing or Error at passing. There was a main effect of congruency on peak velocity [*F*(1, 6) = 6.19, *p* = 0.047, *ηp*^2^ = 0.51] with slightly higher peak velocities in incongruent (120.7 ± 13.6 cm/s) than congruent (119.2 ± 13.4 cm/s) conditions. This shows a slightly different velocity profile depending on congruency, but note that no effects were found on movement time.

As expected, the results of the control experiment do not mirror those of the main experiment. This suggests that the increase in crossmodal interaction when participants’ hands neared the non-target in the main experiment, seen only in the visuotactile condition, indeed depends on the obstructing nature of the non-target.

### Distance between the hand and the non-target

**Fig. 5:**
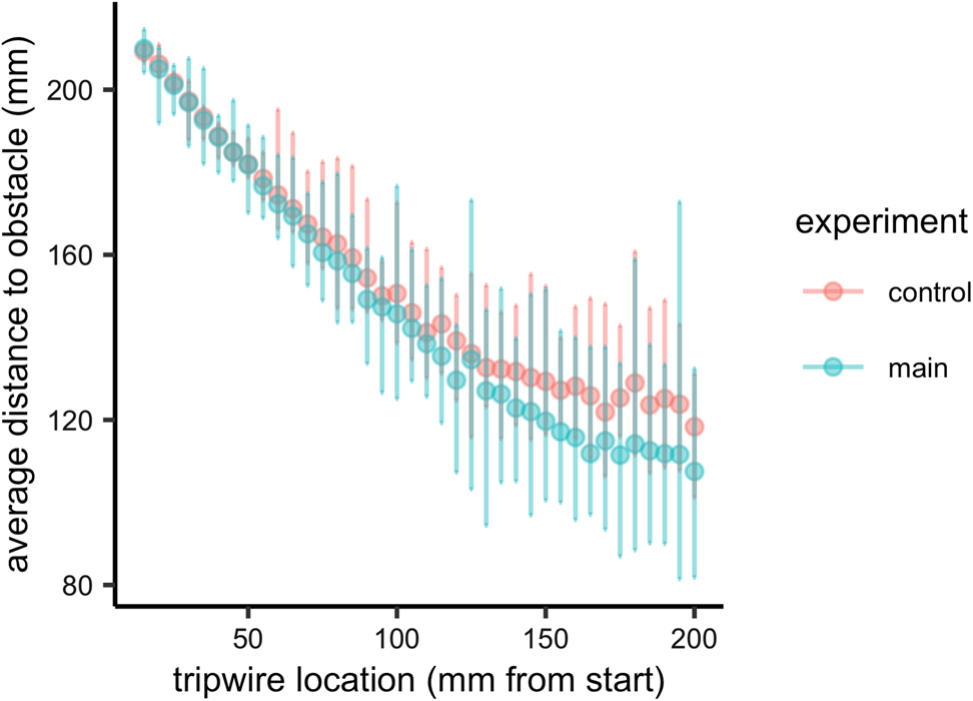
average distance from the index finger to the obstacle at the different tripwire locations. Error bars depict minimum to maximum distance per tripwire location

In the current experiment, the action was always performed with the right hand and non-targets were placed at the same distance from the midline in the main and the control experiment. But because of the asymmetrical nature of hand movements, there were differences between the experiments in the actual Euclidian distance between the hand and the non-target at the time of the trigger (see Fig. 5). This figure nevertheless shows a considerable overlap between the hand-non-target distances for the main and control experiment. We, therefore, re-analysed only those trials of the main experiment in which the hand-obstacle distance fell within the range of the distances observed for that particular tripwire location in the control experiment. We reran the same repeated measures ANOVA with modality and congruency as factors and reaction time as dependent variable. Due to a loss of power because of the limited number of trials that could be included, the interaction between congruence and modality was no longer significant: modality *F*(1, 8) = 0.02, *p* = 0.892, *ηp*^2^ < 0.01, congruency *F*(1, 8) = 3.66, *p* = 0.092, *ηp*^2^ = 0.31, modality*congruency *F*(1, 8) = 2.84, *p* = 0.130, *ηp*^2^ = 0.26. However, as we had a clear hypothesis about the to-be-expected differences, we decided to perform planned *t *tests in which differences between congruent and incongruent conditions were tested separately for the visuotactile and tactile modality. They clearly showed that the congruent–incongruent difference remained significant for the visuotactile modality, but not for the tactile modality: visuotacitle: Paired *t* test: *t*(8) = − 3.57, *p* = 0.007, *d* = − 1.68; tactile only: Paired *t *test: *t*(8) = 0.18, *p* = 0.858, *d* = 0.09. We suggest, therefore, that the difference in hand–obstacle distance cannot fully explain the difference in crossmodal congruency effects between the main and control experiments.

## Discussion

Although there has been increasing interest in how various sensory cues are weighted and integrated to enable a multisensory representation of peripersonal space (e.g. Rizzolatti et al.^1997^; Spence and Driver 2004), this is the first study to investigate this interaction for obstacle avoidance. Our aim was, therefore, to investigate whether predicting the tactile consequences of contact with the object is not only relevant for goal-directed actions but also for avoiding potential collisions with obstacles. In this study, participants performed reaches towards a target object, while a to-be-avoided non-target object was also present in the workspace. During this action participants were subjected to tactile stimulation or visuotactile stimulation. Moreover, this stimulation could be congruent or incongruent: the stimuli were presented on the same side of the acting hand or on opposite sides of the acting hand. We used the spatial location of the hand to trigger stimulation in order to carefully map participants’ responses across space. Importantly, movement tracking was used to determine the participants’ kinematics.

The data showed interactions between modality and congruency, suggesting a cross-modal congruency effect (CCE). We found that that visuotactile responses were less accurate, particularly in the bimodal incongruent condition. Also, reaction times over all tripwire locations showed faster reaction times in congruent compared to incongruent conditions. Critically, and as we hypothesised, the reaction times depended on the distance between the hand and the obstacle, but only in the bimodal congruent condition. Recalculating this measure as a difference in reaction time between congruent and incongruent conditions (CCE) also suggested that multisensory interactions increase when a hand nears an obstacle. As revealed in a control experiment, these effects were only present when the non-target object actually obstructed the movement.

So, we demonstrate that multisensory interactions increase when a hand nears an obstacle. This is a novel demonstration as, so far, there have been demonstrations of increased visuotactile interactions during goal directed grasping, when a hand nears a to-be-interacted-with object (Brozzoli et al. 2010; Patane et al. 2019), or at the end of a grasping movement, when a secondary action needs to be performed (e.g. grasp candy and bring it to your mouth), but only when the end goal of the secondary movement is plausible (candy fits in mouth) (Senna et al. 2019). Our data extend the results by Brozzoli et al. (2010) who were the first to show cross-modal congruency effects increase when actors’ hands approach a target object. These authors also showed differential modulations of peripersonal space for two different actions, namely pointing and grasping. Crucially, Brozzoli et al. (2010) demonstrated that grasping triggered enhanced cross-modal interactions as the actor’s hand neared the terminus of action compared to pointing. So, these enhancements occur only when the target object was to be interacted with. We replicate their data with our experiment: we also show an increased cross-modal interaction as the hand nears the obstacle. Apparently, avoiding obstacles fits along the continuum set by pointing and grasping. In fact, when speculating about where obstacle should fit along that line, we consider that obstacle avoidance (CCE 1th quantile 43 ms, 3rd quantile 92 ms) might be closer to Grasping (CCE between 20 and 80 ms as reported by Brozzoli et al. (2010); CCE 40–110 ms as reported by Patane et al. 2019) than pointing (CCE between 20 and 50 ms as reported by Brozzoli et al. (2010)). Furthermore, our experiment offers two important extensions. First, we have used space in order to trigger visuotactile stimuli instead of time. Whereas Brozzoli and colleagues (2010) probed cross-modal interaction prior to, at the start of, and during action execution, we probed cross-modal interaction at 40 different locations. This means that the resolution of our experiment was higher. In addition, our continuous design allowed us to quantify the relationship between space and the cross-modal congruency effect. This way, we can effectively point to a relation between space and the CCE, or rather between space and cross-modal interactions underlying the peripersonal space.

In the current experiment, we show increased multisensory interactions when the object is to be avoided, that is, when it is relevant for but not the terminus of action. This result redefines the automatic and flexible nature of online peripersonal space remapping during movements. The results suggest that during voluntary movements, the anticipated tactile consequences of contact with a nearby object are automatically evoked, even when contact with the object is not planned. We conclude that the anticipatory multisensory-motor interface between the body and environment also takes collisions into account when driving any voluntary action. Our data firmly support the posited functions of the peripersonal space (PpS) (Rizzolatti 1997; Brozzoli et al. 2014): in order to successfully control the movement trajectory of a hand around obstacles (and towards a goal), the PpS incorporates current visual stimuli and predicted tactile consequences. Above all, this is in order to move efficiently and to protect the manipulandum (e.g. a hand) from harm by anticipating the negative consequences of actions.

Although the CCE has seen widespread use as an index for measuring the multisensory representation of peripersonal space (see Maravita et al. 2003 for a review), there are two alternative explanations for the disparate performances by participants during congruent and incongruent trials. First, it is possible that purely the competition between responses elicited by a target stimulus and a distractor stimulus affects processing times. That is, on incongruent visuotactile trials, the tactile stimulus is the target stimulus, whereas the visual stimulus is the distracting stimulus. The distracting effect on incongruent trials is mainly due to the disparity between the locations of the visual and the tactile stimulus. Both the tactile and visual stimuli evoke responses associated with the side on which they are presented. Given that the distractor activates the wrong response, and that this response needs to be inhibited before the correct response can be given, slower responses to the target will occur (see Tipper et al. 1997 for a detailed discussion of the mechanism underlying this process) than in situations where the stimuli are presented on the same side. In that case, the performance by people on congruent trials might show response facilitation, since the target and the distractor stimuli would both activate the same, “correct” response.

Second, the slower performance by participants during trials with incongruent stimuli might reflect a perceptual interaction between vision and touch, similar to the ventriloquism effect reported by Bertelson and de Gelder (2004). In this case, one stimulus is mislocalised toward the other, i.e. the tactile stimulus is mislocalised (closer) to the visual stimulus location (or vice versa). Possibly, the reason for this is the phenomenon of tactile suppression (see, e.g. Juravle et al. 2013). Tactile suppression is the inhibition of tactile perception during movement execution. Coupled with the idea posited by Alais and Burr (2004) that for bimodal stimulation localization the more reliable stimulus dominates localization, it may be that in our experiment the visual stimulus dominates the tactile stimulus which leads to a mislocalisation of the tactile stimulus toward the visual stimulus. This means that it would be more difficult for people to determine the true location of the tactile stimulation for incongruent stimuli compared to congruent stimuli, resulting in longer processing times for incongruent stimuli. Conversely, the tendency toward localizing visual and tactile stimuli to one spatial location may also speed up congruent responses, because -in this case- the locations are already closer together. According to Spence et al. (2004b), however, such (mis)localizations may “account for only small components of the overall cross-modal congruency effects reported” (p. 166). We, therefore, also consider the contribution of the ventriloquism effect to the CCE as relatively small. Thus, we consider the response competition explanation for the CCE, where the competition evoked by two conflicting signals needs to be overcome, the most likely.

One caveat is in order when interpreting response competition effects; both the labels facilitatory and inhibitory may be attributed to the effects of congruent and incongruent stimulation. That is, we can both argue that congruent stimuli facilitate processing and that incongruent stimuli inhibit processing, while the observed effect remains the same: a difference in measured response times between conditions. In all, this subtle nuance means we can only point to differences between the bimodal congruent and incongruent conditions, instead of proclaiming facilitatory or inhibitory effects. The main point of the discussion yet remains: the cross-modal congruency effect increases when the actor’s hand comes closer to the non-target.

Taken together, we consider that the results of this cross-modal congruency study on obstacle avoidance behaviour generate promising leads: first of all, we have quantified visuotactile remapping relative to distance for obstacle avoidance behaviour, so the boundaries of remapping (e.g. radial or cardinal) could be further explored. Secondly, since reaching to grasp while avoiding obstacles combines both hypothesized functions of the PpS, which are defending the organism from harm and guiding voluntary action, it would be interesting to explore the relative hierarchy of these functions.
